# Effectiveness of Dental and Maxillary Transverse Changes in Tooth-Borne, Bone-Borne, and Hybrid Palatal Expansion through Cone-Beam Tomography: A Systematic Review of the Literature

**DOI:** 10.3390/medicina57030288

**Published:** 2021-03-19

**Authors:** Giovanni Coloccia, Alessio Danilo Inchingolo, Angelo Michele Inchingolo, Giuseppina Malcangi, Valentina Montenegro, Assunta Patano, Grazia Marinelli, Claudia Laudadio, Luisa Limongelli, Daniela Di Venere, Denisa Hazballa, Maria Teresa D’Oria, Ioana Roxana Bordea, Edit Xhajanka, Antonio Scarano, Felice Lorusso, Alessandra Laforgia, Francesco Inchingolo, Gianna Dipalma

**Affiliations:** 1 Department of Interdisciplinary Medicine, University of Bari Aldo Moro, 70124 Bari, Italy; giovanni.coloccia@gmail.com (G.C.); ad.inchingolo@libero.it (A.D.I.); angeloinchingolo@gmail.com (A.M.I.); valentinamontenegro@libero.it (V.M.); assuntapatano@gmail.com (A.P.); graziamarinelli@live.it (G.M.); c.lauda@hotmail.it (C.L.); luisanna.limongelli@gmail.com (L.L.); daniela.divenere@uniba.it (D.D.V.); denisahazballa@gmail.com (D.H.); mtdoria51@gmail.com (M.T.D.); alessandra.laforgia@uniba.it (A.L.); francesco.inchingolo@uniba.it (F.I.); giannadipalma@tiscali.it (G.D.); 2 Kongresi Elbasanit, Rruga: Aqif Pasha, 3001 Elbasan, Albania; 3 Department of Medical and Biological Sciences, University of Udine. Via delle Scienze, 206, 33100 Udine, Italy; 4 Department of Oral Rehabilitation, Faculty of Dentistry, Iuliu Hațieganu University of Medicine and Pharmacy, 400012 Cluj-Napoca, Romania; 5 Department of Dental Prosthesis, Medical University of Tirana, Rruga e Dibrës, 1001 Tirana, Albania; editxhajanka@yahoo.com; 6 Department of Innovative Technologies in Medicine and Dentistry, University of Chieti-Pescara, 66100 Chieti, Italy; ascarano@unich.it

**Keywords:** palatal expanders, constricted maxillary arches, teeth malpositions, tomographical studies

## Abstract

*Background and Objectives*: Palatal expansion is a common orthodontic technique able to increase the transverse changes for subjects with constricted maxillary arches. The aim of the present investigation was to evaluate through a systematic review the tomography effectiveness of different palatal expander approaches. *Materials and Methods*: The database used to perform the screening and determine the eligibility of the clinical papers was PubMed (Medline). *Results*: The database search included a total of 284 results, while 271 articles were excluded. A total of 14 articles were included for the qualitative assessment. *Conclusions*: The effectiveness of the present studies reported that skeletal expansion was a useful approach to increase the transverse changes for subjects with constricted maxillary arches.

## 1. Introduction

Transverse maxillary deficiency is a sign of the abnormal, lesser development of the maxillary bone and surrounding structures. It can be caused by poor nasal breathing, trauma, and congenital anomalies, and is usually associated with dental crowding, crossbite, Class II and III malocclusion [1], and also with disorders in the temporomandibular joint [2,3,4]. The prevalence of posterior crossbite is estimated to be 8% to 23% of the population [5].

The size of the upper jaw changes as it grows, and the size and shape of the dental arches vary at different times of the dentition. The anthropometric data described by various authors show that the natural changes of the arches are determined in part by a tooth movement, and in another part by a skeletal growth. The growth of the palatal width is usually determined from infancy to adolescence, and the intercanine and intermolar distance increases significantly from birth up to 13 years and then decreases slightly upon completion of the development of the permanent dentition and remains constant [6]. The sample size was composed of untreated subjects that did not need treatment.

Transverse maxillary constriction has to be treated as soon as possible to stimulate the right growth of the maxillary complex and to stimulate the right growth of the mandible [7]. The first and the best orthodontic therapy of transverse maxillary constriction is the application and activation of a palatal expander. Maxillary expansion can be obtained by the application of heavy forces to open the midpalatal suture and to promote maximal bony repositioning and minimal movement of the dentoalveolar structures [8,9].

The necessity of palatal expansion depends on the discrepancy between the upper and lower arch and the necessity of creating space to align the teeth. Adkins et al. [10] have demonstrated that every millimeter in transpalatal width increase in the premolar region produces a 0.7 mm increase in the available maxillary arch perimeter; so, if it is necessary to solve a skeletal posterior crossbite or dental crowding, palatal expansion has to be considered. It is also necessary to consider that maxillary transversal constriction is often associated with sagittal discrepancy common in Class III malocclusion. In skeletal Class III malocclusions, it is sometimes needed to apply an extraoral facemask appliance to protract the maxilla in association with the palatal expander to expand the maxilla not only transversally but also sagittally.

**Figure 1:**
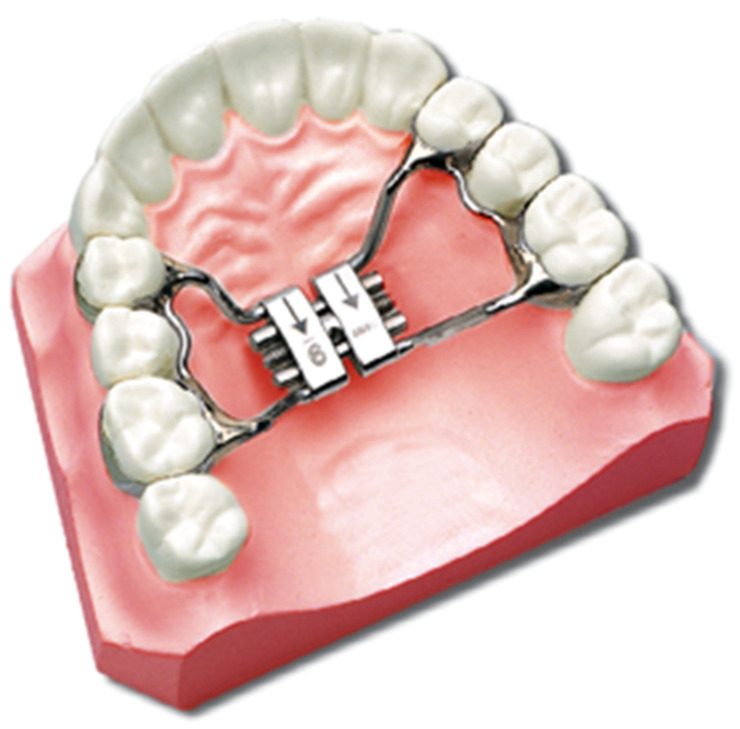
Graphical representation of the palatal expander.

The palatal expander has evolved greatly during the last years. From the first publication by Angell in 1860 [11], rapid maxillary expansion has been the most common orthodontic therapy to increase the maxillary transverse width (Figure 1).

It has also been demonstrated to be a procedure with few complications [12], and was proven to be effective in obstructive sleep apnea (OSA) [13] treatment in children [14]. Based on the speed of expansion due to the number of activations in a certain treatment time, maxillary expansion techniques can be divided into rapid maxillary expansion (RME) and slow maxillary expansion (SME). SME or RME have no significative differences on the clinical outcome of treatment of patients with posterior crossbite [15]. Both RME and SME showed moderate evidence of similar short-term dentoalveolar effects with smaller skeletal expansion than the dentoalveolar. Long-term alveolar effects are supported by moderate evidence for RME and skeletal long-term effects are reported only with RME with very low evidence [16]. Based on the speed of expansion due to the number of activations in a certain treatment time, maxillary expansion techniques can be divided into rapid maxillary expansion (RME) and slow maxillary expansion (SME) [16]. In particular, RME has been described as an activation of the midpalatal screw of 0.5–1 mm/day and SME has been described with an activation of 0.25 mm/day. It seems that SME is a more physiological way to expand, because it is associated with constant expansion of both teeth and bone, while RME is characterized by a more rapid bone expansion but with later relapse of half of the initial bone expansion [16].

Usually, first maxillary permanent molars or second maxillary temporary molars are used for anchorage of the maxillary expander. The expander can be activated by discontinuous forces according to a rapid or slow protocol or by continuous forces via a pre-activated Ni-Ti expander [17,18]. The skeletal expansion of the midpalatal suture is possibly due to the hyalinization of the periodontal ligament of the anchored teeth and the transmission of the forces to the maxilla [19,20,21,22].

However, anchorage teeth have been demonstrated to move during expansion, and that means that a part of the expansion is dental and another part is skeletal [23]. Dental expansion can be considered as a side effect of tooth-borne rapid or slow expansion. For that reason, Logiudice et al. in 2018 have investigated the periodontal negative effects, such as teeth tipping and buccal bone decrease, in growing patients after tooth-borne expansion (evaluated with cone-beam computed tomography (CBCT)), finding in all cases molar buccal tipping and a reduced thickness of the molar buccal bone [24]. Maxillary skeletal expansion with teeth anchorage is effective in growing patients. Due to the increasing resistance of the midpalatal suture to the expansion, a surgical approach with weakening of the midpalatal suture before tooth-borne expansion (SARPE) is desirable in adults [25]. Maxillary expansion evolved in the last years. Lee in 2010 [26] first described the effectiveness in the palatal expansion of a hybrid miniscrew and teeth anchored expander (MARPE) in a single case report of a 20 year old. Hybrid-anchored expansion, with two miniscrews and first upper molars anchorage, did not show the undesirable effect of excessive dentoalveolar expansion and was therefore considered as an alternative method to SARPE in late adolescents who need to have skeletal expansion. After 10 years, a recent meta-analysis confirmed that mini-implant assisted rapid expansion (MARPE) could decrease the loss of buccal alveolar bone when compared to conventional tooth-borne palatal expansion [27,28,29,30,31].

The author assessed that palatal expansion supported by miniscrews (MARPE) represents an alternative to increase orthopedic changes and avoid undesirable effects in late adolescence and adulthood [27]. Recent studies have shown better results using MARPE expanders compared to tooth-borne expanders, even in skeletally mature patients [32,33]. Since its introduction in 1998, cone-beam computed tomography (CBCT) [34] has been widely used in dentistry to help diagnoses and treatment planning. Despite its usefulness, the risk of unnecessary exposure to ionizing radiation has to be prevented [35]. According to three systematic reviews, 2D or 3D X-rays should be performed only to give additional information in the diagnosis, or implementation of the treatment plan, or to evaluate progress or complications during treatment [36,37,38]. The use of CBCT in orthodontics is always indicated in cases where there are impacted teeth, labiopalatoschisis, and skeletal dysmorphism that require maxillofacial surgery, supernumerary intervention [39].

CBCT allows to have a series of information on hard tissues that allows measurements with a low margin of error. 

CBCT imaging can be useful to evaluate constricted airways in OSA patients and to check the efficacy of RME and surgery as treatment options [40,41,42]. CBCT has the opportunity to give better information than 2D images or dental cast studies, in particular to evaluate bone thickness and teeth movement before and after maxillary expansion [43].

In particular, CBCT has been precisely used to give information about how expansion can affect periodontal and dental structures and how expansion determines teeth movement [44,45,46].

Finally, CBCT has been made safe and simple to plan mini-implant placement in every orthodontic case that required skeletal anchorage [47,48]. The aim of this research is to describe the bone and dental effects, assessed by cone-beam computed tomography (CBCT), of the expansion of maxilla due to different orthodontic appliances: a bone-borne expander, tooth-borne expander, or hybrid-borne expander. In particular, this research wants to analyze the ratio between the width expansion of the bone and teeth at the level of the first molars.

## 2. Materials and Methods

### 2.1. Search Strategy

The present investigation was conducted according the Cochrane Handbook for Systematic Reviews of Interventions [49] and reported in accordance to the Preferred Reporting Items for Systematic Reviews and Meta-Analysis (PRISMA) guidelines [50]. The systematic review was also performed according to the STROBE [51] statement for observational studies reports. The paper screening was conducted independently by to expert reviewers (F.I. and G.C). Any disagreement was resolved by discussion with a third author (F.V.). The paper identification was conducted using the PubMed database, to find the articles concerning the effects of bone-borne and hybrid-borne skeletal maxillary expansion on alveolar bone and teeth assessed with CBCT, published in English up to December 2020, for the last 10 years.

### 2.2. Selection of Studies

The following inclusion criteria were selected to assess the eligibility of the studies: related human clinical studies; studies conducted on patients with maxillary deficiency; studies conducted with treatment with a skeletal (bone-borne) or hybrid maxillary expander; and non-surgical maxillary expansion therapy (Table 1). From the initial abstract screening, the studies with cone beam tomography findings were included for the eligibility evaluation. Articles including patients with previous orthodontic treatment, periodontal disease, endodontic treatment of posterior teeth, tooth agenesis, with anomalies, or with congenital syndromes, were excluded.

The authors screened all the titles and abstracts retrieved from the databases and reviewed the full texts of the potentially relevant studies. The level of agreement between the two reviewers was assessed by Cohen’s kappa statistics. 

### 2.3. Data Collection

The extraction of the study data was performed according to the study design, sample size, age, sex, skeletal maturity, orthodontic appliance, activation protocol, width changes of maxilla and teeth, follow up, and the skeletal and dental outcomes of the included studies at the first upper molar level. Moreover, the CBCT tomography dental width changes were extracted from the papers included. The patients were treated with expansion using tooth, hybrid, and bone-borne expanders; we thus considered all the appliances for skeletal expansion.

## 3. Results

### Selection of Studies and Study Characteristics

**Figure 2:**
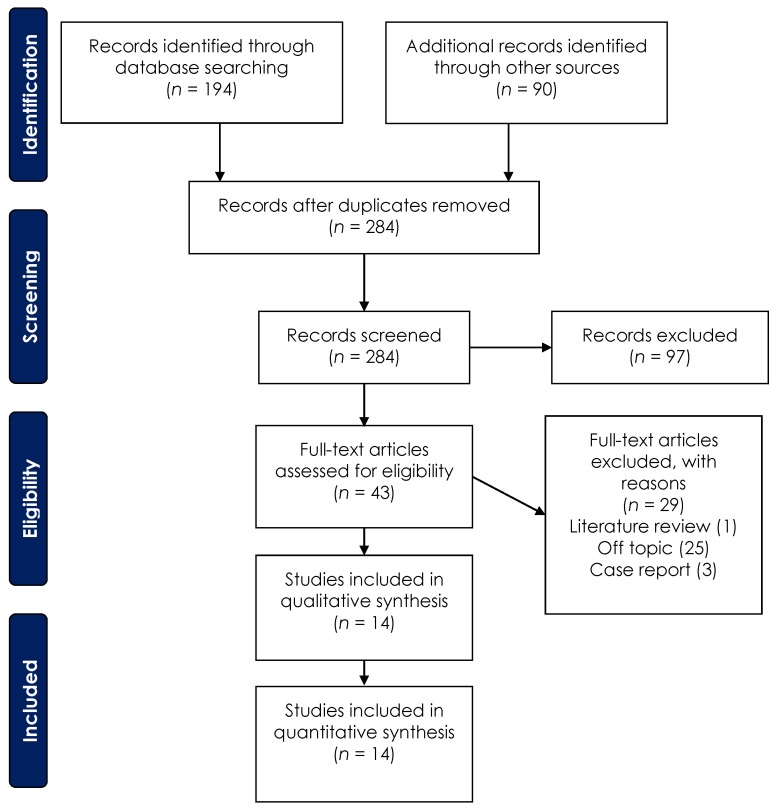
PRISMA (Preferred Reporting Items for Systematic Reviews and Meta-Analysis) flow diagram depicting the selection of the eligible studies.

The electronic database search identified a total of 194 items and 90 articles were included through a manual search. The papers considered for the abstract screening was 284 items. In total, 97 articles were excluded after the abstract screening and 43 papers were considered for the eligibility assessment. Subsequently, 29 papers were excluded after the full text evaluation: 25 off-topic and non-tomography studies, 1 literature review, and 3 case reports. Finally, 14 articles were considered eligible for this systematic review (Figure 2).

Ten of them are retrospective studies, while four of them are randomized, controlled clinical trials. All the selected studies evaluated dental and skeletal effects after expansion with and without bone anchorage provided by miniscrews. The sample size of the studies ranged from 15 to 60, and the mean age ranged from 9.3 years to 22.6 years. Only one article described skeletal maturity, which was determined by the method of the cervical vertebral maturation (CVM) [52] to be over CS 4. In one study [53], the patients were divided into two groups, depending on their age. In the first group, the patients were 8 to 16 years old, and in the other group the patients were aged older than 16. The appliances used were traditional dental-supported expanders, hybrid (tooth- and miniscrew-supported) expanders, and just miniscrew-supported expanders. Just two studies [54,55] had a control group. Eight studies, which included all the randomized clinical trials (RCTs) [33,54,55,56,57,58,59,60], compared the dental and skeletal effects of the traditional RME with the tooth-borne or hybrid expander.

The activation protocols were almost the same in the ten studies [54,55,57,58,59,60,61,62,63,64] but the amount of expansion was different for all studies because it depended on the amount of skeletal discrepancy. All the studies described the end of the activations when the skeletal discrepancy was overcorrected with occlusal contacts between the palatal cusps of the upper posterior teeth and lingual cusps of the posterior lower teeth. 

All the patients in the considered studies underwent a CBCT examination before treatment (T0). The observation period was different between the selected studies. In particular, two studies [64,65] observed patients after 3 weeks or 38 days after expansion, while five studies [33,56,57,62,63] performed CBCT after 3 months. Five [54,55,58,60,61] studies performed the CBCT examination after 6 months, and one [53] after 7.8 months on average. Only three studies [54,59,60] showed effects after one or more years. Data about the dental and skeletal effects at the first molars level were extracted after reading all full-text articles and are reported in Table 2 and Table 3. Two articles did not provide data about the dental effects, just the skeletal effects [61,62].

## 4. Discussion

**Figure 3:**
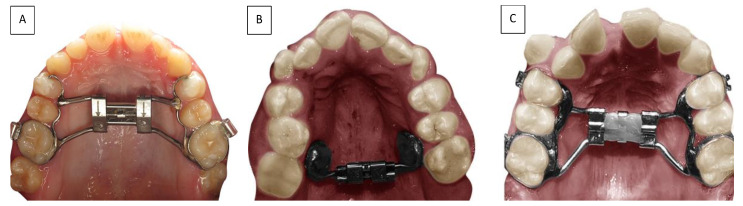
Different palatal expansion appliances: (**A**) the hybrid expander with skeletal and tooth anchorage; (**B**) the bone-borne expander with full skeletal anchorage; (**C**) the conventional expander with teeth anchorage.

In this study, our aim was to analyze data about the amount of bone and dental expansion in full bone-borne and hybrid expanders (Figure 3), assessed by CBCT in the last 10 years. The selection of articles that fitted the selection criteria was difficult and the data of the studies were very heterogeneous. For that reason, we decided to include also observational studies and not only RCTs. In particular, it was difficult to extract the dental and bone expansion values because not all the studies used the same landmarks. Important differences were found about the age of the patients and the time of observation. 

The limits of the present assessment are with regard to the wide tomography measurement heterogeneity while only the data about bone and dental expansion at the first molar level were considered for the qualitative analysis. No data about dental casts were extracted in the presence of CBCT data.

All studies selected showed different but all positive short-term results about the dental and skeletal effects of expansion. Paredes et al. analyzed the different effects on the right and left sides of the maxilla, and the ratio between the skeletal effect, alveolar bending, and dental tipping during maxillary expansion with pure skeletal anchorage by four miniscrews [64]. The author observed that the skeletal expansion determines a skeletal midpalatal expansion with skeletal bending, with the center of rotation located at the most external and inferior point of the zygomatic process of the frontal bone or slightly above and parallel to the interfrontal distance [64]. This study showed that the traditional linear method underestimates the orthopedic expansion effect and overestimates dentoalveolar effects, and suggests to find at first the center of rotation of the maxillary bone, which can be different between appliances and protocols, and measure the angle determined with the other anatomic structures [64]. The angular values showed a ratio of skeletal expansion, alveolar bending, and dental tipping of, respectively, 96.58% (skeletal expansion on the right side), 95.44% (on the left side), 0.34% (alveolar bone bending on the right side), 0.33% (on the left side), 3.08% (dental tipping on the right side), and 4.23% (on the left side) [64]. When comparing traditional RME with tooth-borne or hybrid expanders, and the control group, Mehta [54] assessed that, in the short term, both showed similar expansion, but in the long term of 2 years and 8 months follow up, the bone-borne expander led to a significant increase in palatal width compared to the RME and control groups. In this study, the mean age was over 13 years. We know that RME is more effective in younger patients. As mentioned, Paredes [64] analyzed the different effects on the right and left sides of the maxilla, and the ratio between the skeletal effect, alveolar bending, and dental tipping during expansion with pure skeletal anchorage by four miniscrews. This study made angular and linear evaluations of the bone and dental effects, showing an important difference between them. The author assessed that the skeletal expansion determines a skeletal bending that alters the linear measurement, and the angular values showed a ratio of 96.58% skeletal expansion on the right side, 95.44% on the left side, 0.34% alveolar bone bending on the right side, 0.33% on the left side, 3.08% dental tipping on the right side, and 4.23% on the left side.

Bazargani [60] conducted an RCT study on patients aged between 9.3 y and 9.5 y that compared dentoalveolar and skeletal effects of tooth-borne and hybrid expanders with 1 year follow up. He concluded that skeletal expansion in the midpalatal suture was significantly higher in the hybrid expander group, but not clinically significant. Dental expansion, alveolar bending, and tipping of the molars after 1 year were not different between the groups. This author states also that the tooth-borne expander does its job in young preadolescent patients, but the hybrid expander can be more effective in upper airway obstruction. 

Lagrèvare [55] conducted a RCT with follow up of 6 months comparing the effects of an RME, full bone-borne expander, and control in 13.5-year-old patients. He concluded that the bone-borne expander produced a lower component of dental expansion compared to RME, while both appliances showed similar levels of skeletal expansion. 

Like Lagravère, Yi [63] stated that the tooth-borne expander can produce more transverse bone expansion, relieve maxillary transverse deficiency, and improve upper airway ventilation in a group of patients that was aged more than 19 years on average, but these results were found after just 3 months. 

Like Yi, Li [62] found good skeletal results after 3 months of treatment, and the patients were aged more than 22 years on average. Differences between the two studies were found also in the type of appliance, which was a full skeletal anchored expander in Yi’s research and a hybrid (four miniscrews and first molars) anchored expander. Park [65], in a group of patients slightly more than 20 years old on average, treated with an hybrid appliance with anchorage at the first molars, first premolars, and four miniscrews, showed that the expansion was performed with little tipping of the molars and little loss of buccal bone thickness. 

In almost all studies that compared skeletal and dental effects between RME with full skeletal or hybrid anchorage expansion therapy [33,54,55,56,57,58,59,60], more dental expansion than skeletal expansion was found in RME therapy than skeletal or hybrid anchored expansion therapy with short-term observation. On the contrary, skeletal expansion was more in the bone-borne expander than RME expansions. That means that the side effects of expansion, such as dental movement and buccal inclination of molars, which can cause periodontal negative effects, are less in bone-borne expansion. 

The direction and amount of expansion described by Cantarella [61] showed that the expansion of the midpalatal suture was parallel to the bone-borne expander. In particular, the split at the anterior nasal spine (ANS) was 4.8 mm and at the posterior nasal spine was 4.3 mm. We know this from a tooth-anchored expansion sample from a patient aged slightly more than 17 years. 

## 5. Conclusions

Due to the recent introduction of bone-borne and hybrid expanders and the recent application of CBCT in dental practice, it is difficult to find studies about the dental and bone effects assessed with CBCT. We have to think about the real necessity for skeletal expansion using more invasive methods, such as bone-borne anchored appliances, in preadolescent patients, because it seems that both tooth-borne expanders have the same skeletal and dental effects. On the contrary, some studies seem to encourage skeletal expansion using bone-borne appliances, with less negative effects such as dental tipping; however, this finding was only in the short term. 

## Figures and Tables

**Table 1:** Electronic database search strategy and keywords

	Search Strategies
Keywords:	Advanced keywords search: ((rapid expander OR hybrid maxillary expander OR non surgical maxillary expansion) AND (retrospective study OR prospective study OR controlled study))
Databases	PubMed/Medline, EMBASE

**Table 2:** Summary of the qualitative analysis of the studies included, regarding the study design, sample size, gender, age, skeletal maturity, and type of appliance.

Author, Year	Study Design	Sample Size (Gender)	Average Age	Skeletal Maturity	Type Of Appliance
Cantarella, 2017	Observational	15 (6 M, 9 F)	17.2 ± 4.2 years	CS > 4	Hybrid expander (4 miniscrew and 1st molars)
Li, 2020	Observational	22 (4 M, 18 F),	22.6 ± 4.5 years	ND	Hybrid expander (4 miniscrew and 1st molars)
Yi, 2020	Observational	13 (10 F, 3 M)	19.61 ± 5.25 years	ND	Bone-borne (4 miniscrews)expander covered resin
Vassar, 2016	Observational	25 (13 F, 12 M)	13.1 ± 2.1 years	ND	Hybrid expander (2 or 4 miniscrews and 1st molars)
Paredes, 2020	Observational	39 (16 M, 26 F)	18.2 ± 4.2 years	ND	Bone-borne (4 miniscrews) expander
Mehta, 2020	Observational	60, RME *n* = 21,	Average age 13.9 ± 1.14 years Bone-borne *n* = 20. Average age 13 ± 1.74 years. control *n* = 19. Average age 13.3 ± 1.49 years	ND	RME vs. bone-borne (2 miniscrews) expander vs. control
Hyung-Wook Moon, 2020	Observational	48, RME *n* = 23 (14 M, 10 F)	Age 19.2 ± 5.9 years. Bone-borne expander *n* = 24 (5 M, 19 F; age 18.1 ± 4.5 years)	ND	RME vs. bone-borne (4 miniscrews) expander
Kavand, 2019	Observational	36, RME *n* = 18 (8 M, 10 F)	Average age 14.4 ± 1.3 years. Bone-borne *n* = 18 (6 M, 12 F; age 14.7 ± 1.4 years)	ND	RME vs. bone-borne (2 miniscrews)expander
Park, 2017	Observational	14 (9 M, 5 F)	Average age 20.1 ± 2.4 years	ND	hybrid expander (4 miniscrew and 1st premolars and 1st molars)
Lin, 2015	Observational	28, RME *n* = 13,	Average age = 17.4 ± 3.4 years; Tooth-borne *n* = 15. 18.1 ± 4.4 years	ND	RME vs. bone-borne (4 miniscrews) expander
Celenk-Koka, 2018	Prospective RCT	40 (/)	(1) RME 12 F, 8 M; average age 13.84 ± 1.36 years; (2) miniscrew RME 13 F, 7 M; average 13.81 ± 1.23 years	ND	bonded RME with occlusal splints vs. bone-borne (4 miniscrew) expander
Davami, 2020	RCT	29, RME *n* = 14, Dresden *n* = 15	ND	ND	RME vs. Dresden expander
Bazargani, 2020	RCT	52, 2 groups (1) RME TB = 26, 13 males, 13 females	Average age 9.3; (2) BB = 26. 13 males, 13 females; average 9.3 years	ND	RME vs. hybrid (2 miniscrews and 1st molars)
Lagravère, 2020	RCT	50, RME *n* = 17 (9 F, 8 M)	Average age 213.7 ± 1.1 years; bone-borne *n* = 17 (10 F, 7 M), average age 14.1 ± 1.6 years; control *n* = 16 (8 F, 8 M), average age 13.3 ± 1.7 years	ND	RME vs. bone-borne expander vs. control

CS: cervical stages; ND: Not defined; RCT: Randomized clinical trial; TB: tooth-borne; BB: bone-borne, RME: rapid maxillary expansion, M: Male; F: Female.

**Table 3:** Summary of the qualitative analysis of the studies included.

Author, Year	Activation Protocol	Dental Expansion between 1st Molars	Bone Expansion At 1st Molar Level (Mm)	Observation Period
Cantarella, 2017	0.5 mm/day until avg 6.8 ± 1.9 mm	ND	Maxillary expansion at PNS = 4.3 mm, expansion at ANS = 4.8 mm	T0 = before treatment T1 = after 5 ± 2 months
Li, 2020	0.52 mm (4 activations of 0.13 mm) in 1st day, then 0.26 (2 activations of 0.13 mm)/day	ND	1st Molars = 2.00 ± 1 mm	T0 = before treatment T1 = after 3 months expansion
Yi, 2020	0.5 mm/day until expansion of 7 mm	2.16 ± 2.21	1.25 ± 0.69	T0 = before treatment T1 = 3 months after expansion
Vassar,2016	1 mm/day	5.6 ± 2.7	4.2 ± 3.4	T0 = before treatment T1 = avg 7.83 months
Paredes, 2020	0.40 mm/day until avg 8.7 ± 1.2 mm expansion	R = 3.84 ± 1.65 L = 4.17 ± 1.86	R = 2.93 ± 1.16 L = 3.06 ± 1.47	T0 = before treatment T1 = 3 weeks after expansion
Mehta, 2020	2 turns/day	Control: (T1) 42.33 (T2) 42.63 (T3) 45.96 Marpe: (T1) 42.12 (T2) 46.55 (T3) 47.06 RME (T1) 42.38 (T2) 48.45 (T3) 46.72	Control T0 = 22.32 T1 = 22.34 T2 = 23.13 Marpe T0 = 22.21 T1 = 24.47 T2 = 24.13 RPE T0 = 22.82 T1 = 24.29 T2 = 23.86	T0 = before treatment T1 = 6 months, T3 = avg 2 years and 8 months
Hyung-Wook Moon, 2020	RME = 0.20 mm/day BB expander = 0.45 mm/week until separation of suture, then 0.20 mm/day	RME (T1) 4.91 ± 1.50; bone-borne (T1) 4.01 ± 1.42	RME T1 = 2.45 ± 1.37; bone-borne T1 = 2.38 ± 1.35	T0 = before treatment T1 = 3 months
Kavand,2019	0.5 mm/day	maxillary intermolar width at first molar apex level dental (t1)29.1 (t2)32. skeletal (t1)30.5 (t2)32.7 maxillary buccal inclination dental +3°R. 2.3° L skeletal +0.4° R 1.4° L	Palatal width: tooth (t1) 22.9 (t2) 24.4; skeletal (t1) 21.7 (t2) 23.9 dental + 1.5 skeletal 2.2	T0 = before treatment T1 = 3 months
Park, 2017	0.2 mm/day	5.4 ± 1.7	1.7 ± 1.8	T0 = before treatment T1 = avg 38 days
Lin, 2015	Over 7 mm after placement. then 0.25/day	RME= 4.45 ± 1.31; tooth-borne = 3.46 ± 1.06	1st molar RME= 1.14 ± 0.47; tooth-borne = 1.99 ± 1.18	T0 = before treatment T1 = 3 months
Celenk-Koka,2018	2 turns/day avg time 19.7 ± 3.8 days	RME = 4.2 ± 1.7 ; bone-borne = 4.5 ±1.3	RME = 1.1 ± 0.4; bone-borne = 3.1 ± 1.3	T0 = before treatment T1 = 6 months
Davami, 2020	RME = 0.5 mm/day; Dresden = 0.25 mm/day	RME = 4.38 ; bone-borne = 5.28	RME = 1.96 bone-borne = 1.91	T0 = before treatment T1= after avg 2 years
Bazargani, 2020	0.5 mm/day	(T1) RME = 5.2 ±0.4; hybrid = 5.8 ± 0.4 (T2) RME = 3.8 ±0.4; hybrid = 4.1 ± 0.5	(T1) RME = 3.0 ± 0.9; hybrid = 3.5 ±0.8 (T2) RME = 0.3 ± 0.7; hybrid = 0.5 ± 0.4	T0 = before treatment T1 = 6 months T2 = 1 year
Lagravère,2020	0.5 mm/day	RME = 5.19 ; bone-borne = 3.70; control = 0.47	RME = 1.40; bone-borne = 1.51; control = 0.15	T0 = before treatment T1 = after 6 months

ND = no data; avg = average; ND: Not defined; BB: bone-borne, RME: rapid maxillary expansion; RPE: Rapid Palatal Expansion; ANS: anterior nasal spine; R: Right side; L: Left side.

## Data Availability

All experimental data to support the findings of this study are available contacting the corresponding author upon request.

## References

[1] Proffit W.R. (2005). Multicenter, Internet-Based Orthodontic Education: A Research Proposal. Am. J. Orthod. Dentofac. Orthop..

[2] Inchingolo F., Tatullo M., Marrelli M., Inchingolo A.M., Tarullo A., Inchingolo A.D., Dipalma G., Podo Brunetti S., Tarullo A., Cagiano R. (2011). Combined Occlusal and Pharmacological Therapy in the Treatment of Temporo-Mandibular Disorders. Eur. Rev. Med. Pharm. Sci..

[3] Sirbu A.A., Bordea R., Lucaciu O., Braitoru C., Szuhanek C., Campian R. (2018). 3D Printed Splints an Innovative Method to Treat Temporomandibular Joint Pathology. Rev. Chim..

[4] Inchingolo F., Abenavoli F.M., De Angelis F., Orefici A., Santacroce L., Dipalma G. (2017). Conservative Surgical Approach to Restore Necrotic Columella in Patients Undergoing Neonatal Usage of Nasogastric Tube. Ann. Maxillofac. Surg..

[5] Kutin G., Hawes R.R. (1969). Posterior Cross-Bites in the Deciduous and Mixed Dentitions. Am. J. Orthod..

[6] Bishara S.E., Ortho D., Jakobsen J.R., Treder J., Nowak A. (1997). Arch Width Changes from 6 Weeks to 45 Years of Age. Am. J. Orthod. Dentofac. Orthop..

[7] Betts N.J., Vanarsdall R.L., Barber H.D., Higgins-Barber K., Fonseca R.J. (1995). Diagnosis and Treatment of Transverse Maxillary Deficiency. Int. J. Adult Orthod. Orthognath. Surg..

[8] Braun S., Bottrel J.A., Lee K.G., Lunazzi J.J., Legan H.L. (2000). The Biomechanics of Rapid Maxillary Sutural Expansion. Am. J. Orthod. Dentofac. Orthop..

[9] Adina S., Dipalma G., Bordea I.R., Lucaciu O., Feurdean C., Inchingolo A.D., Septimiu R., Malcangi G., Cantore S., Martin D. (2020). Orthopedic Joint Stability Influences Growth and Maxillary Development: Clinical Aspects. J. Biol. Regul. Homeost. Agents.

[10] Adkins M.D., Nanda R.S., Currier G.F. (1990). Arch Perimeter Changes on Rapid Palatal Expansion. Am. J. Orthod. Dentofac. Orthop..

[11] Angell D.H. (1860). Treatment of Irregularity of the Permanent or Adult Teeth. Dent. Cosm..

[12] Haas A.J. (1965). The treatment of maxillary deficiency by opening the midpalatal suture. Angle Orthod..

[13] Budin C., Ciumarnean L., Maierean A., Rajnovean R., Gergely B., Man M., Aluas M., Cozma A., Bordea R. (2019). Therapeutic Alternatives with CPAP in Obstructive Sleep Apnea. J. Mind Med. Sci..

[14] Camacho M., Chang E.T., Song S.A., Abdullatif J., Zaghi S., Pirelli P., Certal V., Guilleminault C. (2017). Rapid Maxillary Expansion for Pediatric Obstructive Sleep Apnea: A Systematic Review and Meta-Analysis. Laryngoscope.

[15] Martina R., Cioffi I., Farella M., Leone P., Manzo P., Matarese G., Portelli M., Nucera R., Cordasco G. (2012). Transverse Changes Determined by Rapid and Slow Maxillary Expansion—a Low-Dose CT-Based Randomized Controlled Trial. Orthod. Craniofac. Res..

[16] Bucci R., D’Antò V., Rongo R., Valletta R., Martina R., Michelotti A. (2016). Dental and Skeletal Effects of Palatal Expansion Techniques: A Systematic Review of the Current Evidence from Systematic Reviews and Meta-Analyses. J. Oral Rehabil..

[17] Beretta M., Lanteri C., Lanteri V., Cherchi C., Franchi L., Gianolio A. (2019). Evolution of the Leaf Expander: A Maxillary Self Expander. J. Clin. Orthod..

[18] Lanteri V., Cavagnetto D., Abate A., Mainardi E., Gaffuri F., Ugolini A., Maspero C. (2020). Buccal Bone Changes Around First Permanent Molars and Second Primary Molars after Maxillary Expansion with a Low Compliance Ni-Ti Leaf Spring Expander. Int. J. Environ. Res. Public Health.

[19] Zhou Y., Long H., Ye N., Xue J., Yang X., Liao L., Lai W. (2014). The Effectiveness of Non-Surgical Maxillary Expansion: A Meta-Analysis. Eur. J. Orthod..

[20] Ballini A., Cantore S., Farronato D., Cirulli N., Inchingolo F., Papa F., Malcangi G., Inchingolo A.D., Dipalma G., Sardaro N. (2015). Periodontal disease and bone pathogenesis: The crosstalk between cytokines and porphyromonas gingivalis. J. Biol. Regul. Homeost. Agents.

[21] Cantore S., Mirgaldi R., Ballini A., Coscia M.F., Scacco S., Papa F., Inchingolo F., Dipalma G., De Vito D. (2014). Cytokine Gene Polymorphisms Associate with Microbiogical Agents in Periodontal Disease: Our Experience. Int. J. Med. Sci..

[22] Cantore S., Ballini A., De Vito D., Abbinante A., Altini V., Dipalma G., Inchingolo F., Saini R. (2018). Clinical Results of Improvement in Periodontal Condition by Administration of Oral Probiotics. J. Biol. Regul. Homeost. Agents..

[23] Garib D.G., Henriques J.F.C., Janson G., de Freitas M.R., Fernandes A.Y. (2006). Periodontal Effects of Rapid Maxillary Expansion with Tooth-Tissue-Borne and Tooth-Borne Expanders: A Computed Tomography Evaluation. Am. J. Orthod. Dentofac. Orthop..

[24] Lo Giudice A., Barbato E., Cosentino L., Ferraro C.M., Leonardi R. (2018). Alveolar Bone Changes after Rapid Maxillary Expansion with Tooth-Born Appliances: A Systematic Review. Eur. J. Orthod..

[25] Angelieri F., Franchi L., Cevidanes L.H.S., Bueno-Silva B., McNamara J.A. (2016). Prediction of Rapid Maxillary Expansion by Assessing the Maturation of the Midpalatal Suture on Cone Beam CT. Dental Press J. Orthod..

[26] Lee K.-J., Park Y.-C., Park J.-Y., Hwang W.-S. (2010). Miniscrew-Assisted Nonsurgical Palatal Expansion before Orthognathic Surgery for a Patient with Severe Mandibular Prognathism. Am. J. Orthod. Dentofac. Orthop..

[27] Copello F.M., Marañón-Vásquez G.A., Brunetto D.P., Caldas L.D., Masterson D., Maia L.C., Sant’Anna E.F. (2020). Is the Buccal Alveolar Bone Less Affected by Mini-Implant Assisted Rapid Palatal Expansion than by Conventional Rapid Palatal Expansion?-A Systematic Review and Meta-Analysis. Orthod. Craniofac. Res..

[28] Comuzzi L., Tumedei M., Pontes A.E., Piattelli A., Iezzi G. (2020). Primary Stability of Dental Implants in Low-Density (10 and 20 Pcf) Polyurethane Foam Blocks: Conical vs Cylindrical Implants. Int. J. Environ. Res. Public Health.

[29] Tumedei M., Piattelli A., Degidi M., Mangano C., Iezzi G. (2020). A Narrative Review of the Histological and Histomorphometrical Evaluation of the Peri-Implant Bone in Loaded and Unloaded Dental Implants. A 30-Year Experience (1988–2018). Int. J. Environ. Res. Public Health.

[30] Maglione M., Bevilacqua L., Dotto F., Costantinides F., Lorusso F., Scarano A. (2019). Observational Study on the Preparation of the Implant Site with Piezosurgery vs. Drill: Comparison between the Two Methods in Terms of Postoperative Pain, Surgical Times, and Operational Advantages. BioMed Res. Int..

[31] Scarano A., Inchingolo F., Murmura G., Traini T., Piattelli A., Lorusso F. (2018). Three-Dimensional Architecture and Mechanical Properties of Bovine Bone Mixed with Autologous Platelet Liquid, Blood, or Physiological Water: An In Vitro Study. IJMS.

[32] Choi S.H., Shi K.K., Cha J.Y., Park Y.C., Lee K.J. (2016). Nonsurgical Miniscrew-Assisted Rapid Maxillary Expansion Results in Acceptable Stability in Young Adults. Angle Orthod..

[33] Lin L., Ahn H.-W., Kim S.-J., Moon S.-C., Kim S.-H., Nelson G. (2015). Tooth-Borne vs Bone-Borne Rapid Maxillary Expanders in Late Adolescence. Angle Orthod..

[34] Hans M.G., Palomo J.M., Valiathan M. (2015). History of Imaging in Orthodontics from Broadbent to Cone-Beam Computed Tomography. Am. J. Orthod. Dentofac. Orthop..

[35] Lukoff J., Olmos J. (2017). Minimizing Medical Radiation Exposure by Incorporating a New Radiation “Vital Sign” into the Electronic Medical Record: Quality of Care and Patient Safety. Perm. J..

[36] Rischen R.J., Breuning K.H., Bronkhorst E.M., Kuijpers-Jagtman A.M. (2013). Records Needed for Orthodontic Diagnosis and Treatment Planning: A Systematic Review. PLoS ONE.

[37] Durão A.R., Pittayapat P., Rockenbach M.I.B., Olszewski R., Ng S., Ferreira A.P., Jacobs R. (2013). Validity of 2D Lateral Cephalometry in Orthodontics: A Systematic Review. Prog. Orthod..

[38] Pittayapat P., Limchaichana-Bolstad N., Willems G., Jacobs R. (2014). Three-Dimensional Cephalometric Analysis in Orthodontics: A Systematic Review. Orthod. Craniofac. Res..

[39] Kapila S.D., Nervina J.M. (2015). CBCT in Orthodontics: Assessment of Treatment Outcomes and Indications for Its Use. Dentomaxillofac. Radiol..

[40] Katyal V., Pamula Y., Martin A.J., Daynes C.N., Kennedy J.D., Sampson W.J. (2013). Craniofacial and Upper Airway Morphology in Pediatric Sleep-Disordered Breathing: Systematic Review and Meta-Analysis. Am. J. Orthod. Dentofac. Orthop..

[41] El H., Palomo J.M. (2014). Three-Dimensional Evaluation of Upper Airway Following Rapid Maxillary Expansion: A CBCT Study. Angle Orthod..

[42] Zeng J., Gao X. (2013). A Prospective CBCT Study of Upper Airway Changes after Rapid Maxillary Expansion. Int. J. Pediatr. Otorhinolaryngol..

[43] Sawchuk D., Currie K., Vich M.L., Palomo J.M., Flores-Mir C. (2016). Diagnostic Methods for Assessing Maxillary Skeletal and Dental Transverse Deficiencies: A Systematic Review. Korean J. Orthod..

[44] Garrett B.J., Caruso J.M., Rungcharassaeng K., Farrage J.R., Kim J.S., Taylor G.D. (2008). Skeletal Effects to the Maxilla after Rapid Maxillary Expansion Assessed with Cone-Beam Computed Tomography. Am. J. Orthod. Dentofac. Orthop..

[45] Rungcharassaeng K., Caruso J.M., Kan J.Y.K., Kim J., Taylor G. (2007). Factors Affecting Buccal Bone Changes of Maxillary Posterior Teeth after Rapid Maxillary Expansion. Am. J. Orthod. Dentofac. Orthop..

[46] Woller J.L., Kim K.B., Behrents R.G., Buschang P.H. (2014). An Assessment of the Maxilla after Rapid Maxillary Expansion Using Cone Beam Computed Tomography in Growing Children. Dental Press J. Orthod..

[47] Abbassy M.A., Sabban H.M., Hassan A.H., Zawawi K.H. (2015). Evaluation of Mini-Implant Sites in the Posterior Maxilla Using Traditional Radiographs and Cone-Beam Computed Tomography. Saudi. Med. J..

[48] Lo Giudice A., Quinzi V., Ronsivalle V., Martina S., Bennici O., Isola G. (2020). Description of a Digital Work-Flow for CBCT-Guided Construction of Micro-Implant Supported Maxillary Skeletal Expander. Materials.

[49] Cochrane Handbook for Systematic Reviews of Interventions. http://handbook-5-1.cochrane.org/.

[50] Hutton B., Salanti G., Caldwell D.M., Chaimani A., Schmid C.H., Cameron C., Ioannidis J.P.A., Straus S., Thorlund K., Jansen J.P. (2015). The PRISMA Extension Statement for Reporting of Systematic Reviews Incorporating Network Meta-Analyses of Health Care Interventions: Checklist and Explanations. Ann. Intern. Med..

[51] Von Elm E., Altman D.G., Egger M., Pocock S.J., Gøtzsche P.C., Vandenbroucke J.P. (2014). The Strengthening the Reporting of Observational Studies in Epidemiology (STROBE) Statement: Guidelines for Reporting Observational Studies. Int. J. Surg..

[52] Baccetti T., Franchi L., McNamara J.A. (2002). An Improved Version of the Cervical Vertebral Maturation (CVM) Method for the Assessment of Mandibular Growth. Angle Orthod..

[53] Vassar J.W., Karydis A., Trojan T., Fisher J. (2016). Dentoskeletal Effects of a Temporary Skeletal Anchorage Device-Supported Rapid Maxillary Expansion Appliance (TSADRME): A Pilot Study. Angle Orthod..

[54] Mehta S., Wang D., Kuo C.-L., Mu J., Vich M.L., Allareddy V., Tadinada A., Yadav S. (2020). Long-Term Effects of Mini-Screw-Assisted Rapid Palatal Expansion on Airway. Angle Orthod..

[55] Lagravère M.O., Ling C.P., Woo J., Harzer W., Major P.W., Carey J.P. (2020). Transverse, Vertical, and Anterior-Posterior Changes between Tooth-Anchored versus Dresden Bone-Anchored Rapid Maxillary Expansion 6 Months Post-Expansion: A CBCT Randomized Controlled Clinical Trial. Int. Orthod..

[56] Moon H.-W., Kim M.-J., Ahn H.-W., Kim S.-J., Kim S.-H., Chung K.-R., Nelson G. (2020). Molar Inclination and Surrounding Alveolar Bone Change Relative to the Design of Bone-Borne Maxillary Expanders: A CBCT Study. Angle Orthod..

[57] Kavand G., Lagravère M., Kula K., Stewart K., Ghoneima A. (2019). Retrospective CBCT Analysis of Airway Volume Changes after Bone-Borne vs Tooth-Borne Rapid Maxillary Expansion. Angle Orthod..

[58] Celenk-Koca T., Erdinc A.E., Hazar S., Harris L., English J.D., Akyalcin S. (2018). Evaluation of Miniscrew-Supported Rapid Maxillary Expansion in Adolescents: A Prospective Randomized Clinical Trial. Angle Orthod..

[59] Davami K., Talma E., Harzer W., Lagravère M.O. (2020). Long Term Skeletal and Dental Changes between Tooth-Anchored versus Dresden Bone-Anchored Rapid Maxillary Expansion Using CBCT Images in Adolescents: Randomized Clinical Trial. Int. Orthod..

[60] Bazargani F., Lund H., Magnuson A., Ludwig B. (2020). Skeletal and Dentoalveolar Effects Using Tooth-Borne and Tooth-Bone-Borne RME Appliances: A Randomized Controlled Trial with 1-Year Follow-Up. Eur. J. Orthod..

[61] Cantarella D., Dominguez-Mompell R., Mallya S.M., Moschik C., Pan H.C., Miller J., Moon W. (2017). Changes in the Midpalatal and Pterygopalatine Sutures Induced by Micro-Implant-Supported Skeletal Expander, Analyzed with a Novel 3D Method Based on CBCT Imaging. Prog. Orthod..

[62] Li Q., Tang H., Liu X., Luo Q., Jiang Z., Martin D., Guo J. (2020). Comparison of Dimensions and Volume of Upper Airway before and after Mini-Implant Assisted Rapid Maxillary Expansion. Angle Orthod..

[63] Yi F., Liu S., Lei L., Liu O., Zhang L., Peng Q., Lu Y. (2020). Changes of the Upper Airway and Bone in Microimplant-Assisted Rapid Palatal Expansion: A Cone-Beam Computed Tomography (CBCT) Study. J. X-ray Sci. Technol..

[64] Paredes N., Colak O., Sfogliano L., Elkenawy I., Fijany L., Fraser A., Zhang B., Moon W. (2020). Differential Assessment of Skeletal, Alveolar, and Dental Components Induced by Microimplant-Supported Midfacial Skeletal Expander (MSE), Utilizing Novel Angular Measurements from the Fulcrum. Prog. Orthod..

[65] Park J.J., Park Y.-C., Lee K.-J., Cha J.-Y., Tahk J.H., Choi Y.J. (2017). Skeletal and Dentoalveolar Changes after Miniscrew-Assisted Rapid Palatal Expansion in Young Adults: A Cone-Beam Computed Tomography Study. Korean J. Orthod..

